# Assessment of Attenuation Coefficient and Blood Flow at Depth in Pediatric Thermal Hand Injuries Using Optical Coherence Tomography: A Clinical Study

**DOI:** 10.3390/ebj6040054

**Published:** 2025-10-01

**Authors:** Beke Sophie Larsen, Tina Straube, Kathrin Kelly, Robert Huber, Madita Göb, Julia Siebert, Lutz Wünsch, Judith Lindert

**Affiliations:** 1 Department of Paediatric Surgery, University Hospital Schleswig-Holstein, 23562 Lübeck, Germany; bekesophie.larsen@uksh.de (B.S.L.); tina.straube@uk-halle.de (T.S.); kathrin.s.kelly@googlemail.com (K.K.); julia.siebert@uksh.de (J.S.); lutz.wuensch@uksh.de (L.W.); 2 Department of Paediatric Surgery, University Hospital Halle, 06120 Halle, Germany; 3 Institute for Biomedical Optics, Universität zu Lübeck, 23562 Lübeck, Germany; robert.huber@uni-luebeck.de (R.H.); m.goeb@uni-luebeck.de (M.G.); 4 Department of Paediatric Surgery, University Medicine Rostock, 18057 Rostock, Germany

**Keywords:** optical coherence tomography, blood flow at depth, attenuation coefficient, pediatric burns, thermal injuries, non-invasive monitoring

## Abstract

Background: Optical Coherence Tomography (OCT) is a high-resolution imaging technique capable of quantifying Blood Flow at Depth (BD) and the Attenuation Coefficient (AC). However, the clinical relevance of these parameters in burn assessment remains unclear. This study investigated whether OCT-derived metrics can differentiate between superficial and deep pediatric hand burns. Method: This prospective, single-center study analyzed 73 OCT scans from 37 children with thermal hand injuries. A structured algorithm was used to evaluate AC and BD. Results: The mean AC was 1.61 mm^−1^ (SD ± 0.48), with significantly higher values in deep burns (2.11 mm^−1^ ± 0.53) compared to superficial burns (1.49 mm^−1^ ± 0.38; *p* < 0.001), reflecting increased optical density in more severe burns. BD did not differ significantly between burn depths, although superficial burns more often showed visible capillary networks. Conclusions: This is the first study to assess both AC and BD using OCT in pediatric hand burns. AC demonstrated potential as a diagnostic marker for burn depth, whereas BD had limited utility. Image quality limitations highlight the need for technical improvements to enhance OCT’s clinical application.

## 1. Introduction

Thermal injuries are a common and serious issue in children, affecting over 30,000 annually in Germany. Around one-third involve the hands [1,2,3,4], often resulting in permanent scarring and functional impairment [5]. Differentiating superficial second-degree burns (Grade IIa) from deeper injuries (Grade IIb/III) remains difficult [6,7]. Clinical guidelines recommend skin grafting if epithelialization does not occur within 2–3 weeks, given the high risk of scarring and the limited accuracy of early clinical assessment. This leads to extended observation and clinical uncertainty. Despite advances such as improved dressings and enzymatic debridement, treatment remains prolonged [1,6,8].

Our research aims to address the ongoing challenge of accurately assessing burn depth in the early stages of injury. While superficial and full-thickness burns can typically be identified with relative certainty, partial-thickness (second-degree) burns remain difficult to evaluate—even for experienced burn surgeons. As a result, clinical management often involves waiting for the wound to demarcate naturally over time.

The potential for spontaneous healing is closely linked to burn depth, particularly the extent of vascular network damage [6]. Therefore, our work focuses on the visualization of tissue injury at various depths, with an emphasis on imaging the vascular network to support early and reliable assessment.

Several imaging techniques are currently under investigation to support clinical decision-making in wound assessment [9,10]. One such method is Laser Doppler Imaging (LDI), which generates color-coded maps of tissue perfusion and has proven effective in distinguishing between burn depths. However, LDI does not provide structural information about the wound [9]. Other optical imaging techniques—such as hyperspectral imaging, multispectral imaging, near-infrared spectroscopy (NIRS), and diffuse reflectance spectroscopy—primarily evaluate tissue oxygenation, offering insights into cutaneous oxygen levels [9,10,11].

Optical Coherence Tomography (OCT) is a promising imaging modality that provides high-resolution (3–5 μm) visualization of the epidermis and the birefringence of dermal collagen fibers to a depth of approximately 2 mm. OCT generates cross-sectional images that can be reconstructed into en face views. Unlike some other imaging techniques, OCT is unaffected by melanin, making it suitable for use across all skin types [10,12,13,14,15,16,17,18,19]. However, its ability to differentiate between tissue types is sometimes limited due to low contrast in conventional OCT images.

An advancement of this technology, Dynamic OCT (D-OCT), utilizes speckle variance from repeated scans to detect signal fluctuations caused by moving erythrocytes, allowing visualization of blood flow and microvascular structures [9,20]. While D-OCT provides valuable functional information, it is highly sensitive to motion artifacts, which can degrade image quality [11].

OCT is already widely used in ophthalmology, with commercial systems routinely employed to visualize retinal and choroidal layers and to diagnose various eye conditions [21,22,23]. Beyond ophthalmology, OCT has been adapted for cardiovascular imaging, particularly in evaluating the morphology of indwelling stents, and is being explored for applications in urology, including the visualization of bladder cancer [19,21].

The most relevant applications of OCT to this study lie in dermatology, where it is used to visualize conditions such as melanomas, basal cell carcinoma, penile lesions, and acne [20,21,24,25,26,27,28,29]. In burn care, multiple research groups are actively investigating the potential of OCT to assess structural and vascular changes in the damaged skin [9,27,28,29]. By observing the application and integration of OCT in clinical pathways, we aim to evaluate its utility in visualizing burn-specific changes in damaged skin [10,12,13] and assessing its potential to accurately determine the severity of burn injuries [10,12,13,18].

Like other research groups, our work is focused on addressing this diagnostic challenge. We aim to enhance clinical evaluation by developing imaging tools that serve as an adjunct to, rather than a substitute for, expert clinical judgment.

Previous work by our group has demonstrated the ability to visualize the involvement of various burn depths [10,13], leading to the proposal of a clinical scoring system [12]. We could confirm the visualization of the vascular structures with OCT. The current study aims to utilize mathematical parameters to develop an objective, user-independent evaluation method.

Building on previous pediatric OCT studies, this work focuses on automatically generated OCT parameters—the Attenuation Coefficient (AC) and Blood Flow at Depth (BD)—and compares them between superficial (Grade IIa) and deep (Grade IIb/III) burns in children.

## 2. Materials and Methods

This prospective, single-center diagnostic study was conducted at the Department of Pediatric Surgery, University Hospital of Schleswig-Holstein (UKSH). OCT scans were acquired from children and adolescents with thermal hand injuries, following a standardized protocol during the first elective dressing change, predominantly 48–72 h post-injury. The study adhered to the Declaration of Helsinki and was approved by the UKSH Ethics Committee, Campus Lübeck (Approval No. 15-116, issued 10 June 2015).

**Figure 1:**
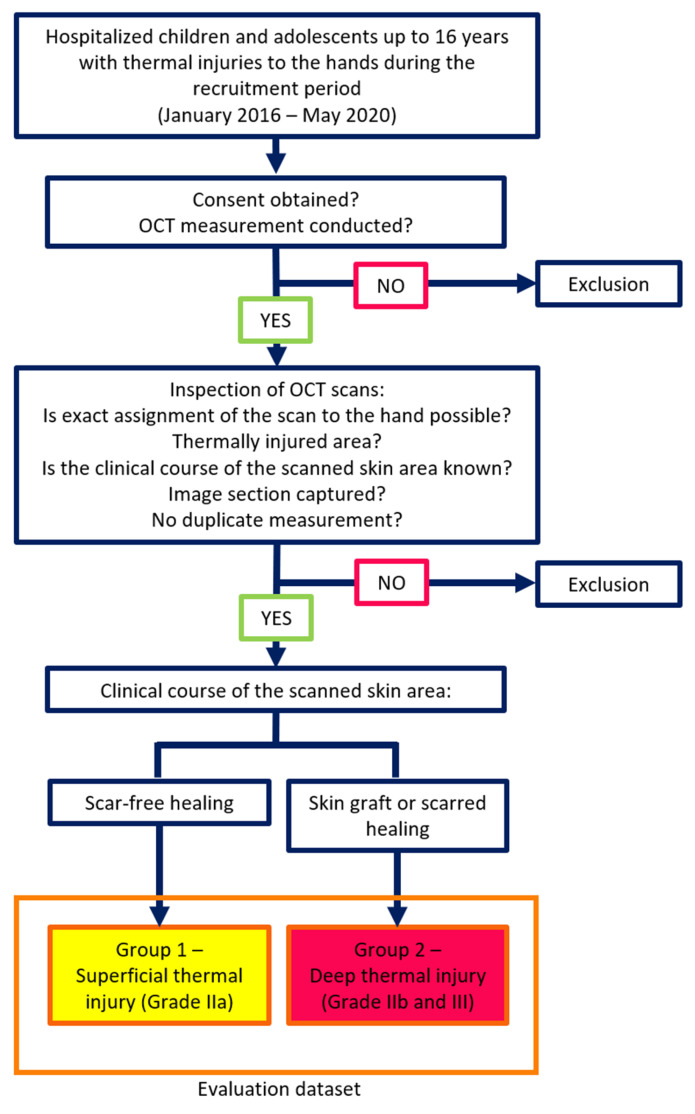
Compilation of the dataset and group assignment.

Patients aged 0 to 15 years treated for thermal hand injuries at the pediatric burn center were included. OCT scans were performed during routine sterile wound care in the operating theatre. The inclusion process is shown in Figure 1.

Each dataset comprised multiple OCT scans and was evaluated for image quality. Quality variation was attributed to anatomical differences, motion artifacts, and the software-generated en face view adjusted to skin curvature. A visual scoring system was developed to objectively assess image quality and support data filtering.

Scans were classified into two groups based on clinical outcomes: Group 1 (superficial burns, Grade IIa) and Group 2 (deep dermal burns, Grade IIb/III).

**Figure 2:**
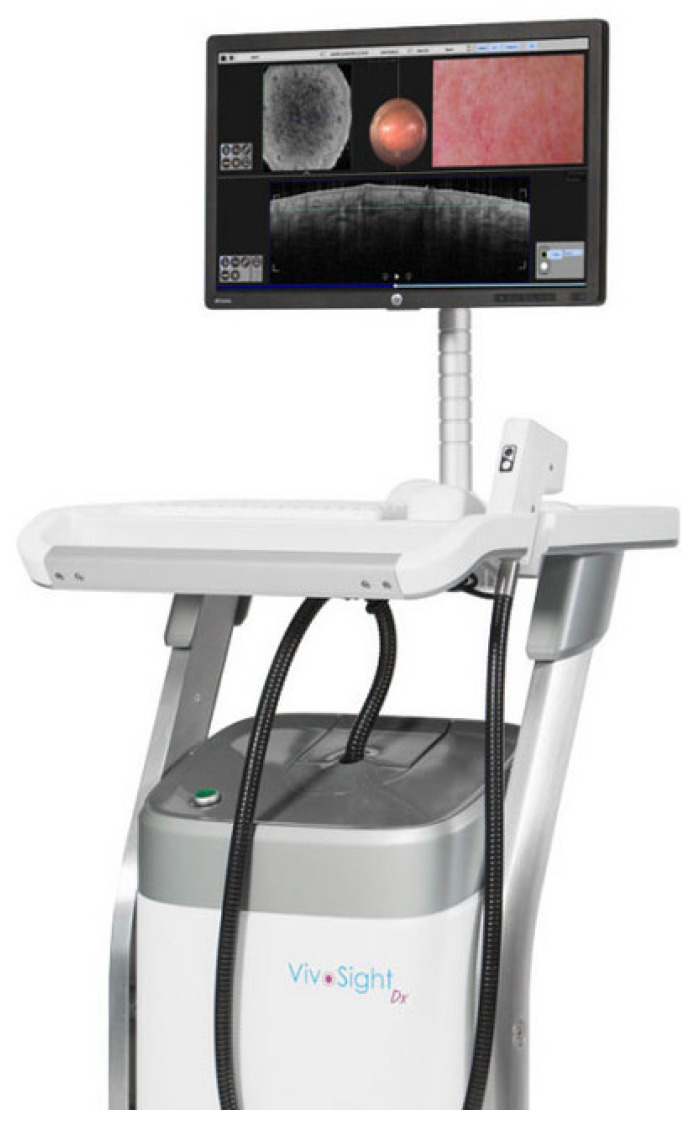
VivoSight Dx 1302© system.

OCT images were acquired using the clinically approved VivoSight Dx 1302© system (Version 4.8, Michelson Diagnostics Ltd., Maidstone, Kent, UK), a swept-source OCT device operating at a 20 kHz A-scan rate with a Class 1 laser at 1305 nm, see Figure 2. Each scan taking 10 s to perform covered a 6 × 6 mm area and penetrated up to ~2 mm, depending on tissue refractive index. The system offers <7.5 µm lateral and <5 µm axial resolution [15,16].

Automated image analysis has become increasingly important in OCT, enabling detection of subtle tissue features beyond the capabilities of visual inspection or standard imaging [17,30,31,32]. In this study, two key parameters—the Attenuation Coefficient (AC) and Blood Flow at Depth (BD)—were extracted using automated software (OCT Analyse, OCT Research Tool, Version 4.0.99.17©, Michelson Diagnostics Ltd.).

In this study, two key parameters—the Attenuation Coefficient (AC) and Blood Flow at Depth (BD)—were extracted using automated software allowing detection of subtle tissue features not visible through direct inspection or conventional imaging (OCT Analyse, part of the OCT Research Tool, Version 4.0.99.17©, Michelson Diagnostics Ltd.).

### 2.1. Attenuation Coefficient

The Attenuation Coefficient quantifies the exponential decrease in OCT signal intensity with increasing depth, caused by scattering and absorption in biological tissue. Lambert-Beer’s law describes this relationship:

*I_z_* = *I*_0_*e*
^−*µz*^

Here, *I*_0_ represents the incident light intensity, *I_z_* is the intensity after a path length *z*, and *μ* is the Attenuation Coefficient. Low values of *μ* correspond to tissue with minimal optical scattering and absorption, while higher values indicate more rapid signal attenuation due to increased optical density.

To ensure accurate measurement, the OCT intensity curve is smoothed to reduce the influence of peaks caused by tissue inhomogeneities. Background noise is removed, and the initial point of the intensity curve is algorithmically determined [30,31].

The calculation, performed by Michelson Diagnostics©, is based on the averaged A-scan from all A-scans within a given OCT stack. A curve-fitting analysis is applied over a depth range of 0.2 mm to 1 mm. The model used for fitting is:

*I_z_* = *A e*
^−2*µz*^ + *Noise*.

In this equation, *A* is a constant determined by *I*_0_ and the sensitivity of the detection system; *μ* is the Attenuation Coefficient, and *Noise* accounts for residual background interference. The factor of 2 reflects the round-trip path of the light (entry and return).

### 2.2. Blood Flow at Depth

Visualizing blood flow in small capillaries is a particular challenge in OCT. Dynamic OCT (D-OCT) addresses this using speckle variance techniques, which detect temporal signal fluctuations caused by red blood cell movement [12,16]. These are displayed as a red overlay, enabling visualization of vascular structures and perfusion dynamics [10,12,33,34].

**Figure 3:**
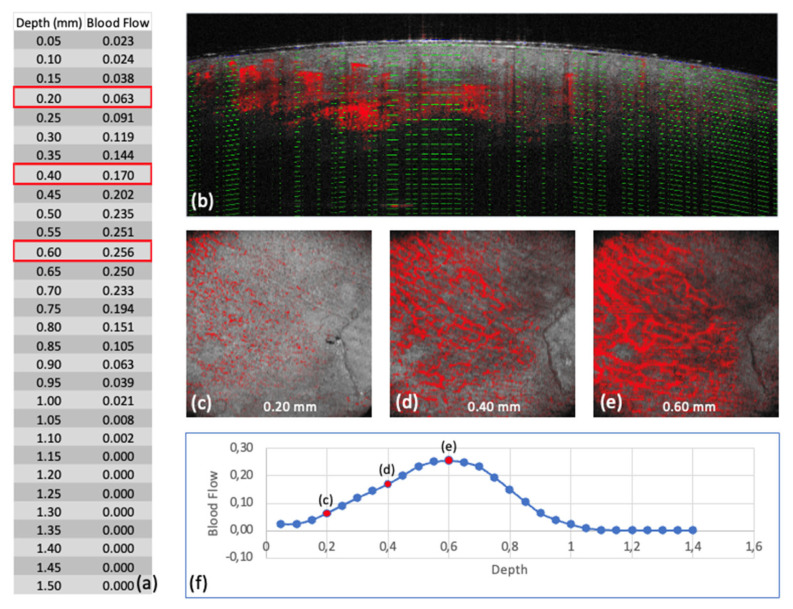
Scan 4 (Group 1—superficial thermal injury (Grade IIa)): (**a**) Output of the Blood Flow at Depth values by the analysis software, with red markings for the depth levels c, d, and e. (**b**) Representation of the Blood Flow at Depth. levels in the B-scan. (**c**–**e**) En face images at different depth levels in Dynamic OCT. (**f**): Graphical representation of the Blood Flow at Depth values, with points representing individual depth levels, and depth levels c, d, and e marked in red.

The Blood Flow at Depth parameter is calculated for each en face depth layer (see Figure 3). The software segments the skin surface and extracts horizontal layers at 0.05 mm intervals. For each layer, the proportion of dynamic signal pixels (ranging from 0 to 1) is calculated relative to the total area.

Notably, the algorithm does not isolate vessels exclusively; signal changes may also reflect tissue motion during acquisition. According to the manufacturer, reliable flow detection is maintained to a depth of 0.5 mm.

We hypothesized that the Attenuation Coefficient and Blood Flow at Depth would differ between superficial and deep thermal injuries, making them valuable diagnostic criteria.

**Figure 4:**
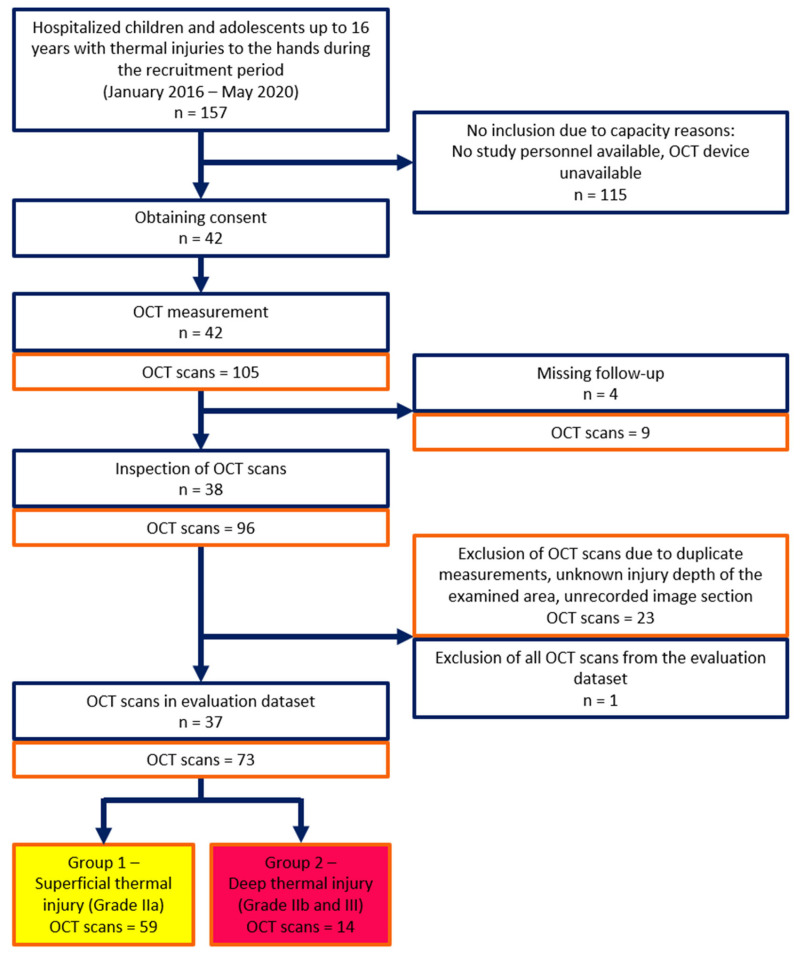
Flow diagram for recruiting the study cohort, study process, and composition of the analysis dataset. Blue outline: Number of children and adolescents. Orange outline: Number of OCT scans.

An overview of the study cohort recruitment, study process, and composition of the analysis dataset, following CONSORT guidelines [35], is provided in Figure 4.

## 3. Results

42 children with thermal hand injuries were scanned with OCT, including children with injuries to both hands. Of these, images of 37 patients (blue), consisting of a total of 73 OCT scan data sets (red), containing several scans for some patients, were included in the analysis. These 73 data sets include 59 OCT scan images of areas clinically allocated to Grade IIa burns, and 14 OCT scan images of Grade IIb and Grade III areas.

**Figure 5:**
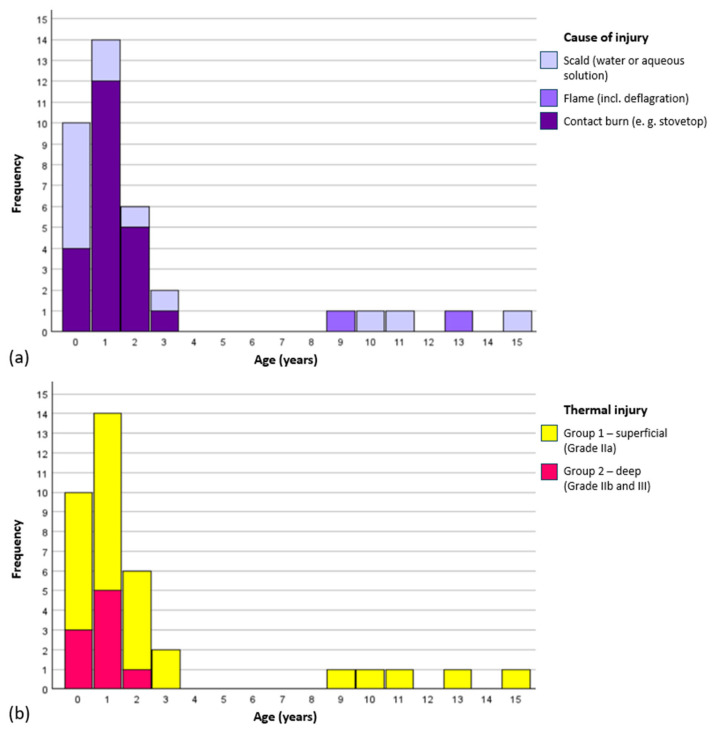
Characteristics of the study cohort (**a**) Cause of Injury, (**b**) Degree of Thermal Injury.

The analyzed scans included children aged 7 months to 15 years, with a predominance of infants and toddlers under 3 years (see Figure 5). The cohort comprised 29 boys and 8 girls. Injuries resulted from 22 contact burns, 13 scalds, and 2 flame burns. Contact burns (stovetops and indoor chimneys) were more common in younger children and often linked to deeper injuries. Despite the reflex to withdraw the hand, the skin is thinner in young children and hence more likely to be seriously affected.

Nine children sustained deeper burns; three healed with scarring, while six required skin grafts. No significant differences were observed between the groups of superficial (Grade IIa) and deep (Grade IIb and III) thermal injuries with respect to age, sex, skin type (glabrous or non-glabrous), time from injury to OCT examination, or OCT image quality. The only significant difference was in the cause of injury. Deep burns resulted exclusively from contact injuries, whereas superficial burns also included scalds and flame injuries (*p* = 0.007).

### 3.1. Attenuation Coefficient

Since both the entire dataset and three subgroups were analyzed, the significance level was adjusted using Bonferroni correction:

α_adjusted_ = α_global_/k = 0.05/4 = 0.0125.

**Figure 6:**
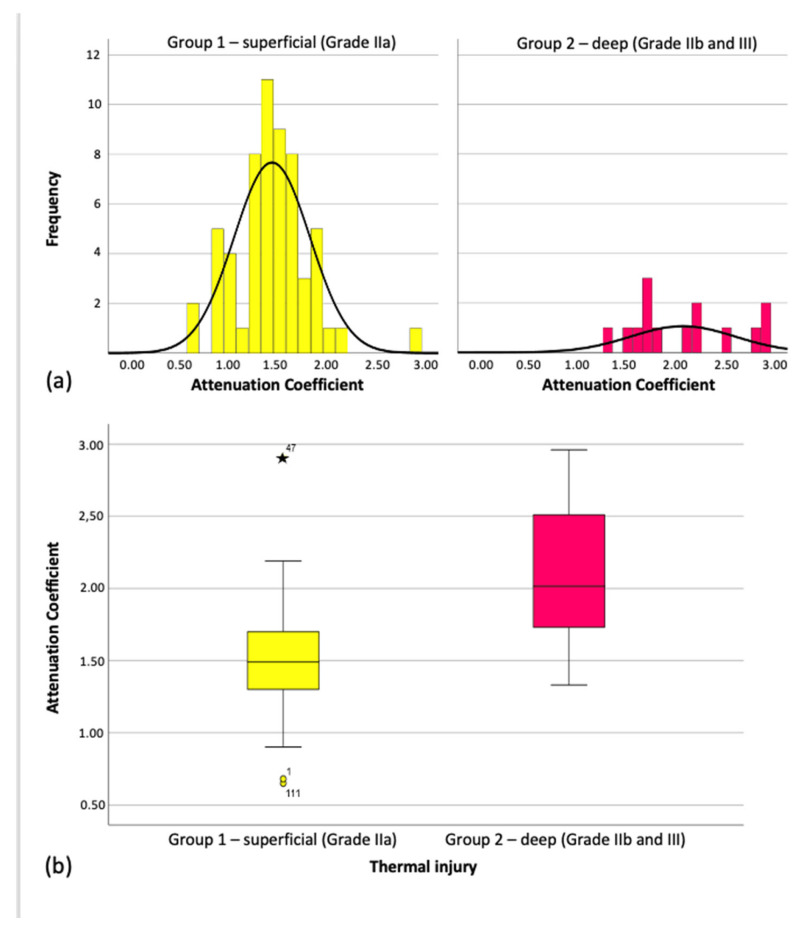
Distribution of the Attenuation Coefficient in the individual groups. (**a**) distribution attenuation coefficient (**b**) boxplot mean attenuation coefficient.

Across all 73 OCT scans, the mean attenuation coefficient was 1.61 mm^−1^ (SD ± 0.48). The overall distribution was non-normal (Kolmogorov–Smirnov: *p* = 0.01; Shapiro–Wilk: *p* = 0.002). However, within individual groups, values appeared normally distributed (see Figure 6), and group means clustered around the overall mean.

On average, the attenuation coefficient for superficial burns (Grade IIa) was 0.62 mm^−1^ lower than for deep burns (Grades IIb/III), a statistically significant difference (Mann–Whitney U = 130.500, *Z* = –3.959, *p* < 0.001). This difference is also reflected in the medians shown in the boxplots (Figure 6) and summarized in Table 1.

#### ROC Analysis

Clinical outcomes and AT cutoff-values were analyzed for sensitivity and specificity. Results were plotted as Receiver Operating Characteristics (ROC) curves.

**Figure 7:**
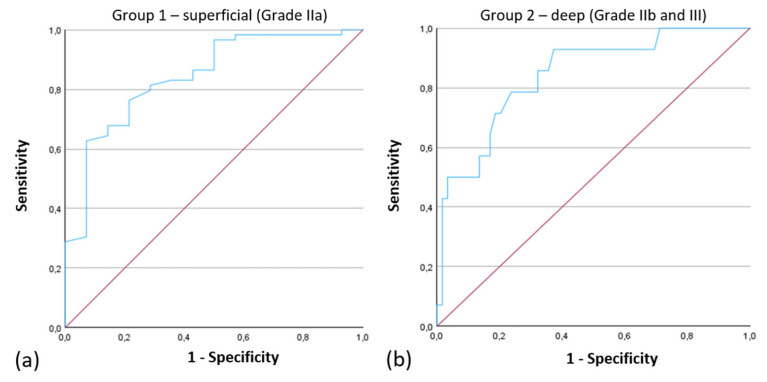
ROC Curves of the Attenuation Coefficient for the Classification into (**a**) “Group 1—superficial thermal injury (Grade IIa)”, Area under the Curve: 0.842 and (**b**) “Group 2—deep thermal injury (Grade IIb and III)”, Area under the Curve: 0.842.

The ROC curve for the Attenuation Coefficient had an Area Under the Curve (AUC) of 0.842, indicating strong performance of the model in distinguishing between Grade IIa and Grade IIb/III injuries (Figure 7).

### 3.2. Blood Flow at Depth

#### 3.2.1. Data Availability Issues

Blood Flow at Depth results were available of 27 OCT images; 21 were from superficial (Grade IIa) and 6 from deep dermal (Grade IIb/III) injuries.

To assess whether this absence might indicate a specific injury depth, the association was analyzed, but no relationship was found (Fisher’s exact test: *p* = 0.759), suggesting that missing values do not correlate with injury depth. Instead, they were associated with image quality. A low image quality score (*p* = 0.003) and a poorly captured field of view (*p* < 0.001) were linked to missing values. Nevertheless, some scans still lacked values despite optimal image quality and a fully captured field of view.

#### 3.2.2. Visual Observations

**Figure 8:**
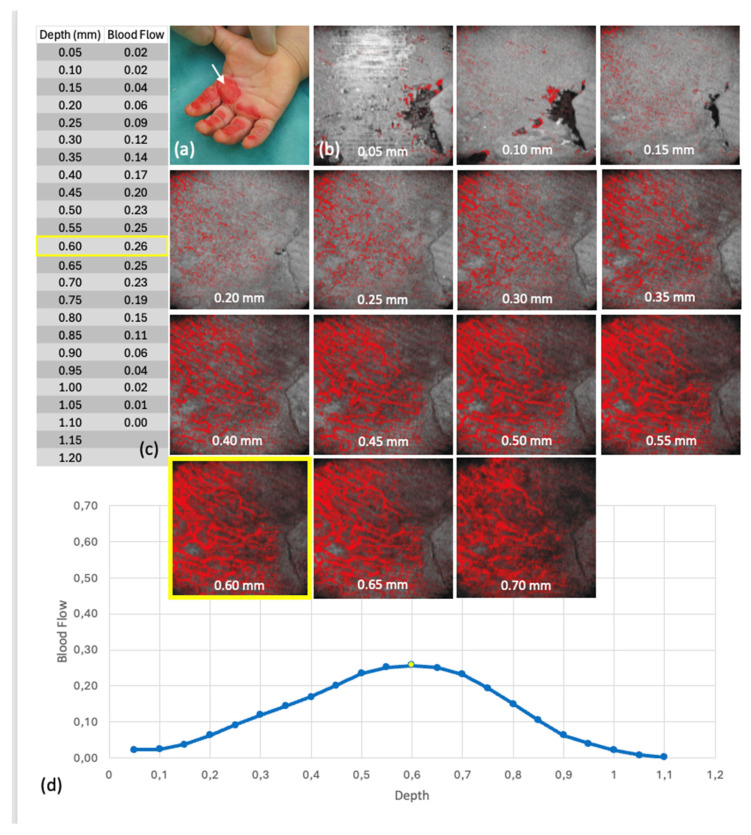
Blood flow at depth from scan 4 (group 1—superficial thermal injury (grade IIa)): (**a**) Clinical findings at the time of OCT, the white arrow points to the area examined by OCT, (**b**) Representation of the en face images belonging to the respective depths of the Blood Flow at Depth in the Dynamic OCT, yellow marking of the maxima, (**c**) Tabulated values of the blood flow at depth, yellow marking of the absolute maximum, (**d**) Graphical representation of the Blood Flow at Depth values, the dots represent the individual levels, the levels of the maxima are marked in yellow.

**Figure 9:**
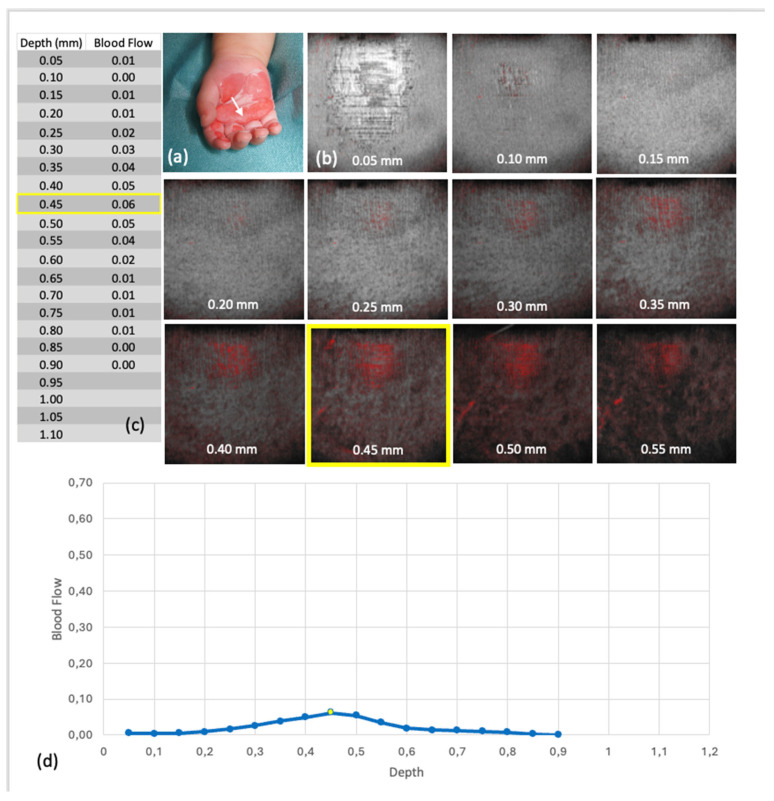
Blood flow at depth from scan 22 (group 1—deep thermal injury (grade IIb/III)): (**a**) Clinical findings at the time of OCT, the white arrow points to the area examined by OCT, (**b**) Representation of the en face images belonging to the respective depths of the Blood Flow at Depth in the Dynamic OCT, yellow marking of the maxima, (**c**) Tabulated values of the blood flow at depth, yellow marking of the absolute maximum, (**d**) Graphical representation of the Blood Flow at Depth values, the dots represent the individual levels, the levels of the maxima are marked in yellow.

In the 27 OCT scans with complete data, visual assessment indicated differences in microvascular structures between superficial and deep burns. Superficial injuries (Grade IIa) often showed more prominent, distinguishable capillary networks, as illustrated in Figure 8 and Figure 9. However, these differences could not be objectively quantified or statistically validated.

#### 3.2.3. Depth-Averaged Blood Flow Values

**Figure 10:**
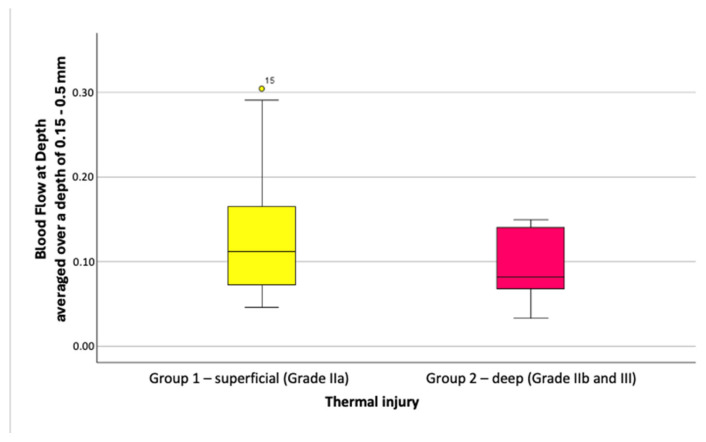
Boxplots of depth-averaged Blood Flow at Depth values, depth range 0.15–0.5 mm (unfiltered dataset).

Blood Flow at Depth values were slightly higher in superficial burns (Grade IIa) than in deeper injuries (Grade IIb/III). At depths of 0.05 to 0.5 mm, blood flow averaged 0.12 ± 0.066 for Grade IIa and 0.09 ± 0.049 for deeper burns, with no significant difference (*p* = 0.408). Similar results were seen at depths of 0.15 to 0.5 mm, with values of 0.13 ± 0.078 versus 0.09 ± 0.045 (*p* = 0.376), as shown in Figure 10.

In summary, depth-averaged blood flow tended to be higher in superficial burns, but this difference was not statistically significant. Filtering for higher-quality images amplified this trend but drastically reduced the sample size (e.g., to three scans per group after excluding scans with motion artifacts).

#### 3.2.4. Quantitative Analysis of Blood Flow Peaks

Maximum blood flow values and related metrics—including the onset of slope before maximum flow, depth at which 50% of maximum was reached, and depth at which 50% of amplitude between maximum and preceding minimum occurred—showed no significant differences between superficial and deep burns. Moreover, the tissue layers where maximum blood flow was measured were often affected by depth-dependent artifacts, limiting their diagnostic value.

### 3.3. Attenuation Coefficient Differences

The study demonstrated that the Attenuation Coefficient significantly differs between superficial and deep thermal injuries. Deeper burns exhibited a notably higher Attenuation Coefficient, indicating increased light scattering and absorption in more severely damaged tissue.

### 3.4. Interpretation of Blood Flow Findings

While Blood Flow at Depth showed apparent differences in microvascular structures between injury groups upon visual inspection of OCT scans, these observations could not be statistically validated or objectively quantified.

### 3.5. Experimental Conclusions

Overall, the Attenuation Coefficient emerges as a promising quantitative marker for assessing burn depth in pediatric thermal injuries. Conversely, Blood Flow at Depth has limited diagnostic utility in this context due to measurement variability and artifact interference.

## 4. Discussion

Optical Coherence Tomography (OCT) offers both high-resolution imaging and quantitative tissue measurements, having a promising potential to aid clinical decision-making in burn management. While its application in burn assessment has been previously explored, limited attention has been given to the underlying tissue optical properties and their clinical relevance—particularly in pediatric populations- Notably, parameters such as the Attenuation Coefficient and Blood Flow at Depth have not been systematically investigated in a well-defined pediatric cohort until now.

Our study cohort reflects well-established epidemiological trends in pediatric hand burns. A significant proportion of injuries (86.5%) occurred in children under the age of four years, consistent with findings from the German Burn Registry and other large-scale epidemiological studies [2,3,4,36]. The mechanisms of injury—primarily scalds, contact, and flame burns—also aligned with registry data, with contact burns being more prevalent in toddlers and often associated with greater depth due to the thin skin morphology in young children. The proportion of deep injuries in our cohort (Grade IIb/III) was 24.3%, in line with previous literature reporting 25–30% [8,36,37].

### 4.1. Attenuation Coefficient

Our findings show a significant increase in the Attenuation Coefficient with burn depth, rising from 1.49 ± 0.38 in superficial burns to 2.11 ± 0.53 in deeper injuries (*p* < 0.001). ROC analysis yielded an AUC of 0.842 for differentiating superficial (Grade IIa) from deep burns (Grade IIb/III), suggesting a potential for the Attenuation Coefficient as a quantitative adjunct to standard visual assessment.

Previous research on this parameter in burns is sparse, often limited to small adult samples or focused on chronic scar tissue rather than acute injuries in children [17,27,31,38]. Differences in OCT systems, calculation methods, and dermal depth definitions complicate direct comparisons. For example, this study used a fixed depth range (0.2–1 mm), while others defined dermal boundaries dynamically [17].

The observed increase in the Attenuation Coefficient is likely driven by structural changes in the dermis following thermal injury. Collagen denaturation, which enhances optical scattering and reflectivity, appears to be the dominant factor. In contrast, superficial burns—characterized by hyperperfusion and edema—alter tissue optical properties in a different manner than deeper injuries with thrombosed vasculature [31,32,39,40,41,42,43,44,45].

### 4.2. Blood Flow at Depth

In Contrary to expectations, Blood Flow at Depth did not significantly correlate with burn severity. High measurement failure rates limited the number of analyzable scans from 73 to just 27, precluding meaningful statistical group comparisons. Nevertheless, these challenges underscore the need for methodological improvements in pediatric OCT imaging.

Despite the technical limitations, visual assessment of OCT scans suggested possible differences in microvascularization between superficial and deep burns. These observations—consistent with deeper vascular involvement or perfusion loss in more severe injuries—could not be objectively validated. Prior studies have shown the utility of Dynamic OCT (D-OCT) in evaluating blood flow under both physiological and pathological conditions, but most have focused on adult subjects or used different imaging systems [19,27,34].

Due to software limitations, Attenuation Coefficient and Blood Flow at Depth were analyzed separately. However, both parameters are affected by common tissue factors, such as vessel density, which can increase forward scattering [31,39]. A combined analysis in future studies may provide a more comprehensive understanding of burn depth and tissue viability.

### 4.3. Strengths and Limitations

This is the first study to evaluate both Attenuation Coefficient and Blood Flow at Depth in a homogeneous pediatric cohort, reducing clinical variability and allowing for more targeted analysis. Scans were performed under anesthesia, simulating realistic clinical conditions and minimizing patient movement. However, this also restricted repeat measurements.

Motion artifacts, inherent to freehand OCT scanning at micrometer resolution, led to the exclusion of a substantial number of scans. The relatively small number of deep burns, along with uncontrolled variables such as comorbidities and body temperature, further limit generalizability. Additionally, the 6 × 6 mm scan area introduces a risk of sampling error, especially in heterogeneous or extensive injuries. Practical aspects of the imaging process, including probe pressure and the use of sterile film covers, may also affect measurements and should be investigated in further studies.

Finally, fixed-depth measurement of the Attenuation Coefficient complicates comparisons between injured and healthy skin with intact epidermis within the same patient.

## 5. Conclusions

This study demonstrated a significant 40% increase in the Attenuation Coefficient with burn depth in pediatric hand injuries, supporting its utility as a diagnostic marker. While Blood Flow at Depth tended to be higher in superficial burns, no statistically significant differences were observed, partly due to motion artifacts and anatomical variability.

To enhance future clinical utility, probe stabilization to reduce motion artifacts and the application of machine learning for automated vascular analysis are recommended. These advances could enable more precise, individualized burn care, improving outcomes and reducing treatment duration.

## Figures and Tables

**Table 1:** Attenuation Coefficient in the individual groups.

Group Assignment	Attenuation Coefficient (mm^−1^)		
	Mean ± Standard Deviation	Min.	Max.
Group 1 (*n* = 59) – superficial thermal injury (Grade IIa)	1.49 ± 0.38	0.65	2.91
Group 2 (*n* = 14) – deep thermal injury (Grade IIb und III)	2.11 ± 0.53	1.33	2.96

## Data Availability

The data presented in this study are available on request from the corresponding author.

## Abbreviations

The following abbreviations are used in this manuscript:

OCT	Optical coherence tomography
BD	Blood Flow at Depth
AC	Attenuation Coefficient
SD	Standard Deviation
UKSH	University Hospital of Schleswig-Holstein
ROC	Receiver Operating Characteristics
AUC	Area Under the Curve
D-OCT	Dynamic OCT
